# Supplementary material for: Decoding of human identity by computer vision and neuronal vision
Source: Sci Rep. 2023 Jan 12;13:651. doi: 10.1038/s41598-022-26946-w (PMC9837190; doi:10.1038/s41598-022-26946-w)
Supplement: Supplementary file 1 — Supplementary Information. [file 41598_2022_26946_MOESM1_ESM.docx]

**Title : Decoding of human identity by computer vision and neuronal vision**

**Authors:** Yipeng Zhang^1,†^, Zahra M. Aghajan^2,†^, Matias Ison^3,†^, Qiujing Lu^1^, Hanlin Tang^4^, Guldamla Kalender^2^, Tonmoy Monsoor^1^, Jie Zheng^4^, Gabriel Kreiman^4,5^, Vwani Roychowdhury^1^, Itzhak Fried^2,6,7^

**Supplementary Figures and Tables:**


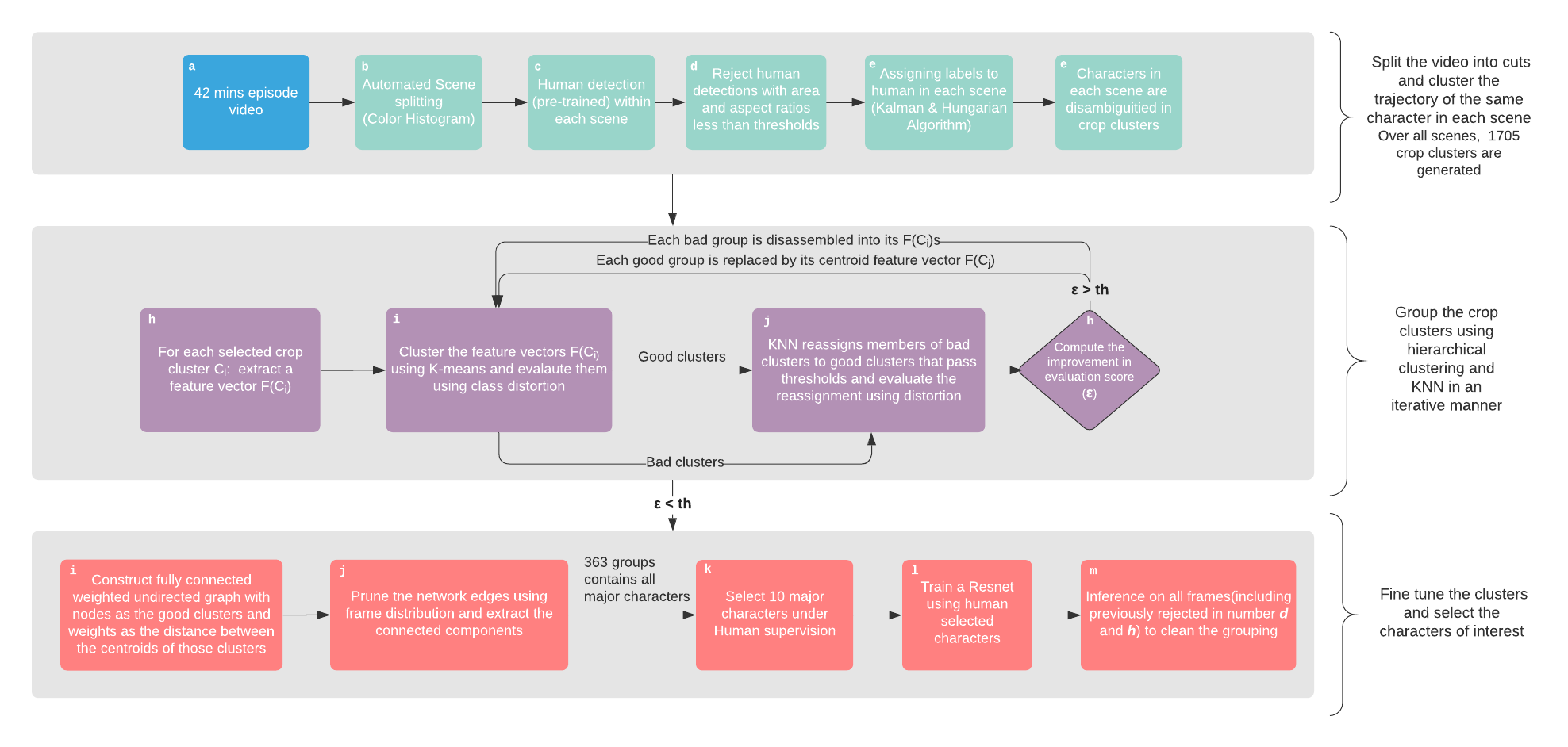


**Supplementary Figure 1.** **Flow chart of Semi-supervised Character Identification.**We developed the semi-supervised framework to identify characters in the movie at the frame level. It consists of three main stages, requiring minimal human supervision but producing reliable ground truth labels to train our decoding models. In the first stage, we split the whole video into cuts based on the scenes (b), then created stable real-time identification of characters, i.e. crop clusters, based on human detection (c,d), and tracking and matching algorithm (e). In the second stage, we merged crop clusters purely based on facial features with k-means clustering (i) and KNN (j) iteratively.


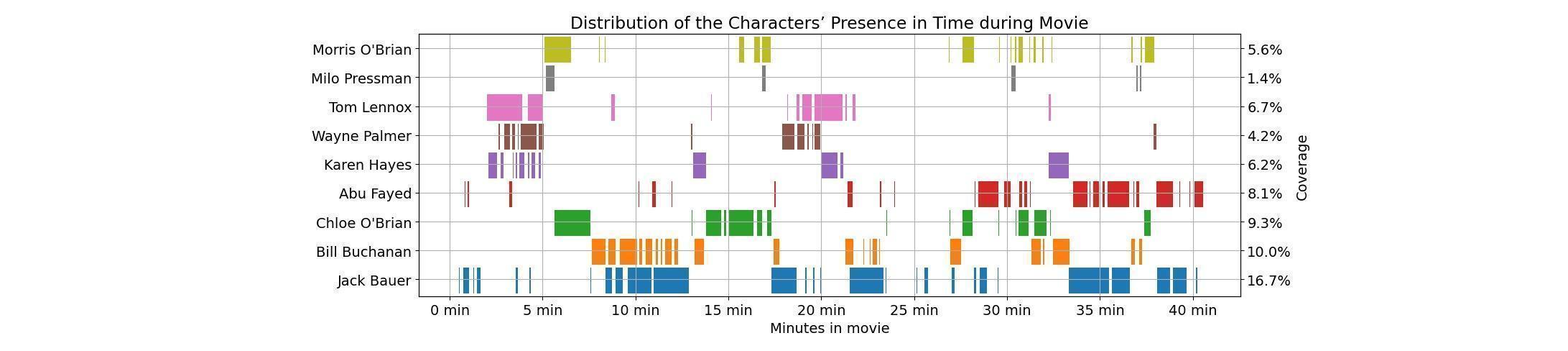


**Supplementary Figure 2.** **Distribution of the Characters’ Presence in Time during the Movie.**The results from our semi-supervised character identification algorithm are shown. The presence of each of the nine characters (rows) in each frame of the movie (x-axis) is indicated by vertical lines. For our subsequent neural decoding analysis, we picked the four characters (bottom 4) that were most prominent and were present during different time points in the movie. For further details about the character distributions in the movie, see Supplementary Table 4. For visualization purposes, each vertical line has been dilated for 50 data points.


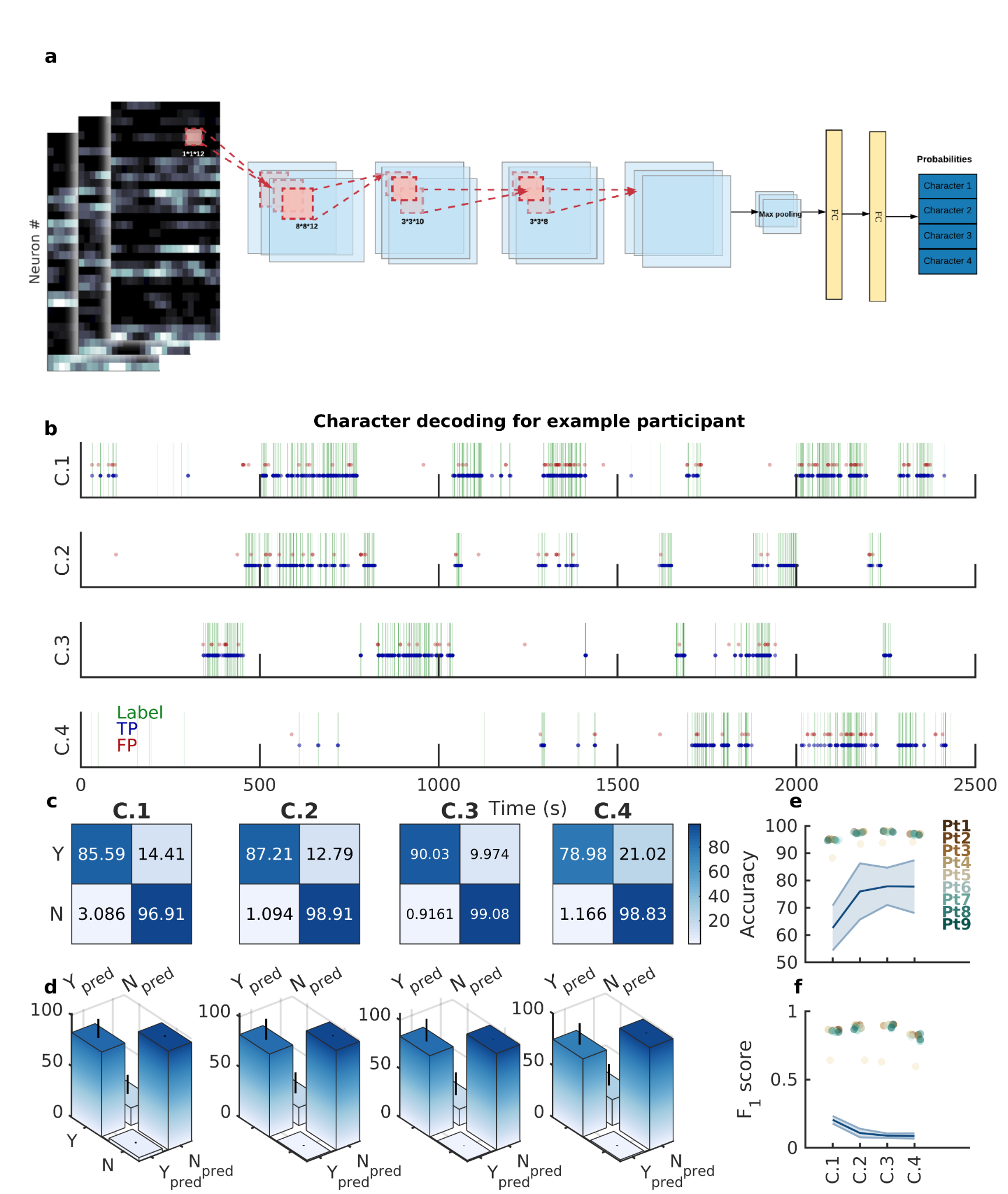


**Supplementary Figure 3.** **Convolutional Neural Network as an Alternative Architecture to Decode Characters from Neural Data (related to Fig. 2).
a.** The structure of the CNN network used for classification. Here, input data was the firing sequence of all neurons (colormap; x-axis: time, y-axis: neuron number; brighter shades correspond to higher firing) from a participant within a two-second window around each frame. Firing rate maps were passed through four CNN layers followed by two fully connected (FC) layers to output a probability distribution over the four main characters in that frame of the movie. **b.** Frame-by-Frame comparison of character labels generated by CV and CNN**:** The labels generated by the CNN and the computer vision algorithm for each movie frame are plotted as a function of time. The significant overlap between the two labels (a large number of true positives) illustrates the goodness of the decoding algorithm (A manual inspection of the time series output of the CNN model for each frame against the true character labels and noted that the model prediction shared high overlap with the true labels). **c.** In an example participant, the normalized confusion matrices for the binary classification task for all the four characters are shown. The large numbers on the diagonals (high TPR and TNR) of all the four matrices show that the CNN achieves high accuracy in decoding all the four characters. **d.** The distribution of the entries of the confusion matrix overall participants is shown as a bar plot (mean) with errorbar (std) for all four characters. The high mean and low standard deviation for the TPR and TNR values in all four matrices shows that the CNN achieves high accuracy in decoding all the four characters across participants. **e-f.** Accuracy (e) and F1-scores (f) for decoding each character are shown with each colored dot indicating different participants. The consistently high accuracy and F1-scores across participants indicate that the CNN generalizes well in this decoding task. The lines and shaded areas (mean±STD) indicate the performance of the chance model (obtained from shuffling labels) across all participants.


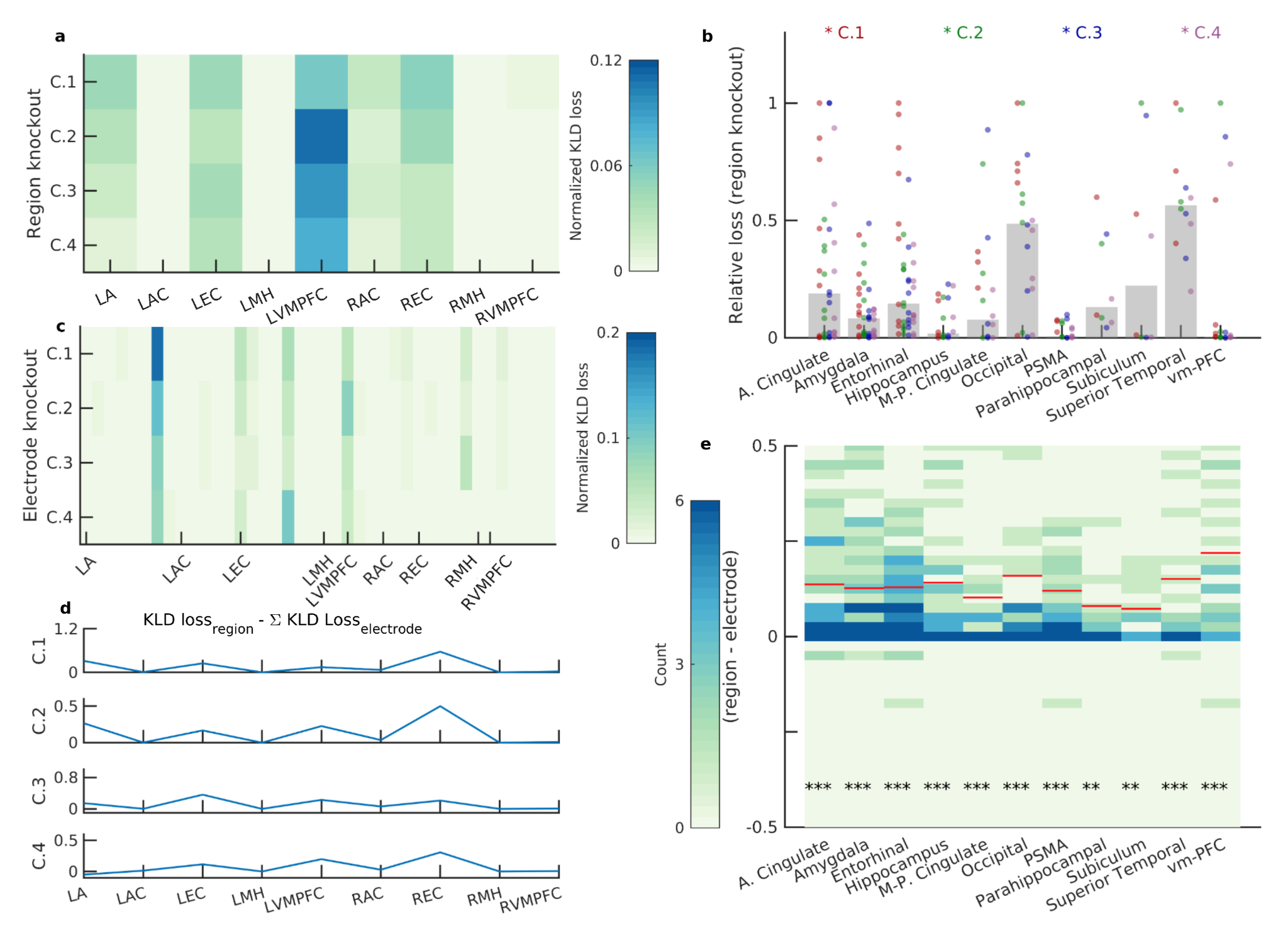


**Supplementary Figure 4. Identification of important regions in decoding characters using a CNN architecture (related to Fig. 3). a.** The change of KLD loss for each character (row) after knocking out a given region (column) for one participant is shown (Region Knockout). The value is normalized by the number of neurons in that region and demonstrates how the model performance deteriorated when excluding the units recorded from that region. Important regions are those with higher KLD loss values. L and R correspond to the left and right hemisphere respectively. A: amygdala, AC: Anterior Cingulate, EC: Entorhinal Cortex, MH: Middle Hippocampus, VMPFC: Ventro-medial Prefrontal Cortex. **b.** The changes in KLD loss after knocking out regions are shown across participants. Different colored dots correspond to the change in KLD loss for different characters. Bars indicate the median value of the change in KLD loss after region knockout. The following regions resulted in the most notable losses in decoding performance: anterior cingulate (22.22, [10.12, 39.15]%), occipital (40.00, [19.12, 63.95]%), Subiculum (37.5, [8.52, 75.51]%), and superior temporal (66.67, [34.89, 90.08]%). Reported are the percentage of losses above 0.5 (as well as the binomial fit confidence intervals) **c.** The change in KLD loss for each character (row) after knocking out a given electrode (column) at a time is shown (Electrode Knockout) for an example participant (same as in a)**.** Similar to the region knockout results in (a), the loss value is normalized by the numbers of units recorded on each electrode. **d.** The sum of the change in KLD loss following electrode knockout (all electrodes within a region) was subtracted from the change in KLD loss following region knockout. Shown are these values for the four different characters (rows) from an example participant (same as in a and c). Positive values indicate that knocking out a whole region deteriorates the model performance to a greater extent. **e**. When considering all regions from all participants, in most regions, the region knockout loss was greater than the sum of electrode knockout loss (each column, and its associated colormap, is the distribution of this measure and the red horizontal line indicates the median of the distribution for those that were significantly different from zero) as quantified by Wilcoxon signed-rank tests (*: p<0.05; **: p<0.01; ***: p<0.001).


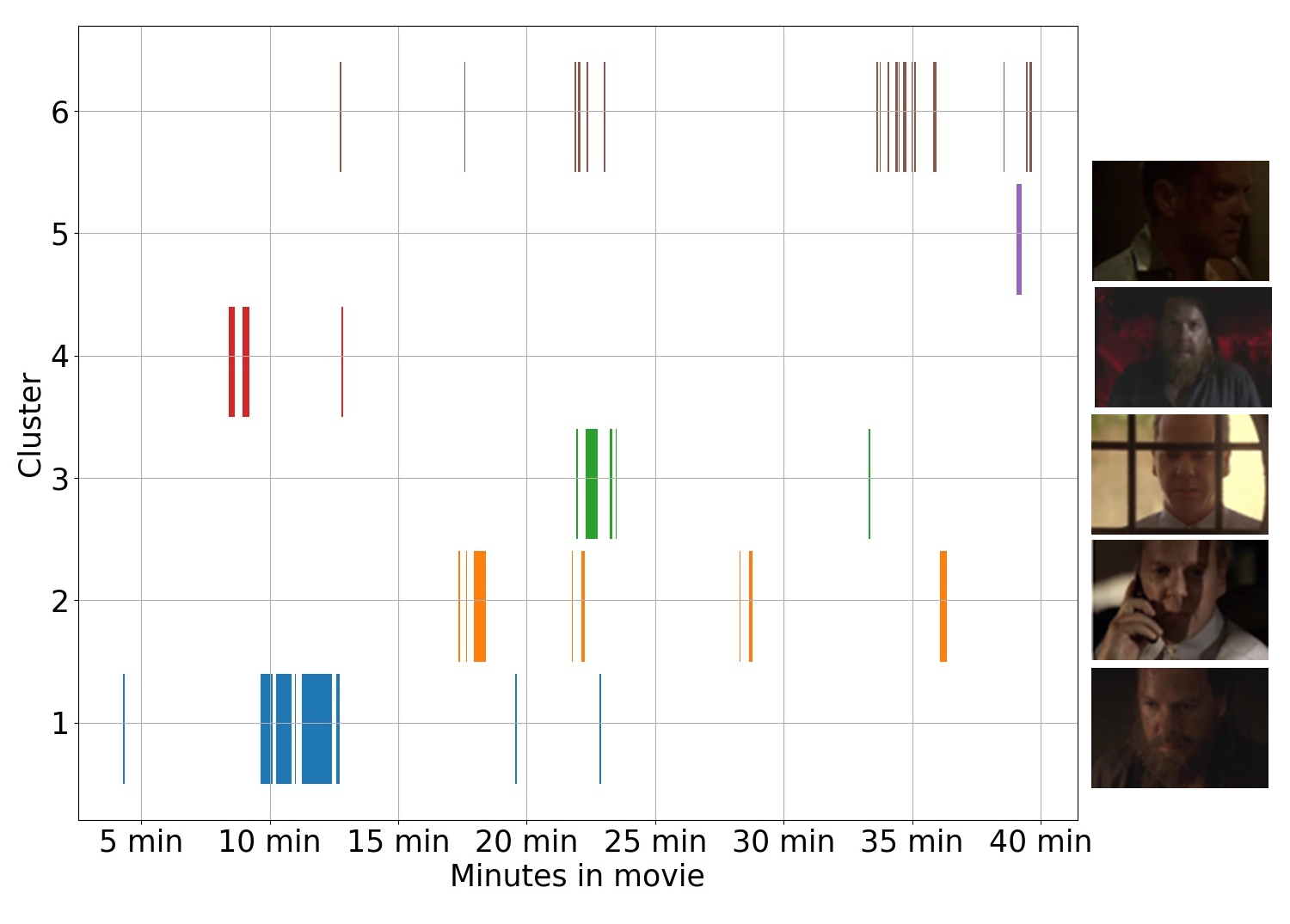


**Supplementary Figure 5. The Semi-supervised Character Identification Algorithm Generated Multiple Clusters for Each Character throughout the Movie.**Our character identification method resulted in multiple clusters for a single character. Each row represents a cluster and the vertical lines indicate the frames in which a given cluster was identified. Note that each cluster corresponds to a visually different representation of the same character (snapshots on the right; cluster six refers to all other small clusters merging together).

| Demographics of Participants | | | |
| --- | --- | --- | --- |
| Pt.  ID | **Age** | **Handedness** | **Gender** |
|  |  |  |  |
| 1 | 50 | R | F |
| 2 | 22 | L | F |
| 3 | 27 | R | F |
| 4 | 31 | R | M |
| 5 | 49 | R | F |
| 6 | 49 | R | M |
| 7 | 24 | R | F |
| 8 | 37 | L | M |
| 9 | 20 | R | M |
|  |  |  |  |

**Supplementary Table 1.** **Participant demographics.**Demographics of the study participants (age, gender, and handedness) are presented.

| **Locs**  **Pt. ID** | **Entorhinal Cortex** | **Hippocampus** | **Para-hippocampal**  **Gyrus** | **Subiculum** | **Amygdala** | **Superior Temporal**  **Gyrus** | **Anterior Cingulate** | **Middle & Posterior Cingulate** | **Ventromedial Prefrontal Cortex** | **PreSMA** | **Occipital** |
| --- | --- | --- | --- | --- | --- | --- | --- | --- | --- | --- | --- |
| **1** | - | L  R | - | - | R | L  R (2) | - | L  R (3) | - | - | - |
| **2** | L  R | - |  |  | - | - | R (2) | R |  | L  R | - |
| **3*** | R | - | L  R | L | - | - | - | - | - | - | L (2) |
| **4** | L  R (2) | L | - | - | L  R | - | L  R | - | L  R | L  R | - |
| **5** | L | - | - | L | L | - | - | - | L | - | - |
| **6** |  | L | R | - | L  R | - | R | - | - | - | - |
| **7** | L | - | - | - | L  R | - | L | - | - | R | - |
| **8** | L  R | L  R | - | - | L | - | L  R | - | L  R | - | - |
| **9** | - | - | - | - | R | - | R | - | - | - | R(2) |

**Supplementary Table 2. Electrode Localizations.**

Electrode locations are listed for each participant (rows). Columns indicate the electrode locations (categories that were used for group analysis) with R and L referring to the right and left hemispheres respectively. The number in parentheses indicates the number of electrodes within each hemisphere. ^*^Participant 3 had units in the parietal region as well.

| **Participant ID** | **Number of Units** |
| --- | --- |
| ***1*** | 56 |
| ***2*** | 53 |
| ***3*** | 49 |
| ***4*** | 84 |
| ***5*** | 8 |
| ***6*** | 18 |
| ***7*** | 29 |
| ***8*** | 60 |
| ***9*** | 28 |

**Supplementary Table 3. Number of Units Recorded in Each Participant.**

|  | **Yes** | **No** | **DNK** |
| --- | --- | --- | --- |
| **C.1** | 17.96 | 80.25 | 1.826 |
| **C.2** | 10.73 | 85.73 | 3.531 |
| **C.3** | 10.01 | 89.76 | 2.230 |
| **C.4** | 8.683 | 89.59 | 1.720 |

**Supplementary Table 4.** **Distribution of the Character Labels during the Movie Generated by the Semi-supervised Framework.**This table shows the percentage of each label for the four main characters detected by our semi-supervised character identification framework on a frame level in the movie. Label Yes (No) was defined as when the character was (not) present at the exact time step (frame). Label DNK (Do Not Know) was introduced as a mechanism to particularize the training set (Methods). The characters were present in less than 20% of the frames across the movie and, thus, it must be borne in mind that the training data used for further neural decoding models were heavily skewed and appropriate measures were taken to remedy this issue. The percentage of DNK was around 2% of the total training samples, which indicates that the dropping of the data sample would not affect the total information provided to the model.

| Layers | | Feature Map | Activation |
| --- | --- | --- | --- |
| Input | Neural Signal | N_neurons_*60 | - |
| 1 | Two-layer LSTM | 128 | - |
| 2 | FC + BatchNorm | 128 | LeakyReLU |
| 3 | FC | 12 | Softmax |

**Supplementary Table 5.** **LSTM Architecture.**The input to the LSTM model consisted of the firing rate of all neurons from a participant in time (2 seconds around each frame with 60 time-steps). This was first fed into a two-layer LSTM followed by a fully connected layer (FC) and a Batch Normalization layer (BatchNorm) with LeakyReLU as the activation function. This was further processed by a connected layer followed by a softmax operation to output the confidence scores for three labels (Yes, No, DNK) for each character.

|  | **Pt. 1** | **Pt. 2** | **Pt. 3** | **Pt. 4** | **Pt. 5** | **Pt. 6** | **Pt. 7** | **Pt. 8** | **Pt. 9** |
| --- | --- | --- | --- | --- | --- | --- | --- | --- | --- |
| **LSTM** | 0.8784 | 0.8766 | 0.8788 | 0.8772 | 0.7597 | 0.8536 | 0.8692 | 0.8793 | 0.8647 |
| **CNN** | 0.8738 | 0.8737 | 0.8633 | 0.8755 | 0.6272 | 0.8620 | 0.8400 | 0.8702 | 0.8483 |
| **NB** | 0.1160 | 0.1144 | 0.1158 | 0.1154 | 0.1180 | 0.1158 | 0.1148 | 0.1137 | 0.1151 |
| **SVM** | 0.1782 | 0.1832 | 0.1901 | 0.1799 | 0.1481 | 0.1523 | 0.1501 | 0.1911 | 0.1573 |
| **LR** | 0.1867 | 0.1503 | 0.1287 | 0.2013 | 0.0000 | 0.0359 | 0.0395 | 0.1682 | 0.0993 |

**Supplementary Table 6.** **Comparing NN Model Performances against a Baseline (Naive Bayes) Model and other ML models.**For each participant (columns), average performance (as quantified by F1-scores) is reported for three different methods used to decode the visual presence of characters using the neural data. Our main methods, the LSTM and the complimentary CNN architectures, fared much (on average 7 times) better when compared against the performance of a Naive Bayes (NB) method, which was used as a baseline model. Similarly, our main NN models outperformed both SVM and Logistic Regression (LR) models in terms of decoding performance.

|  | **Recall** | **Precision** | **F_1_ score** | **Accuracy** |
| --- | --- | --- | --- | --- |
| **C. 1** | 83.59±2.24 | 87.60±2.15 | 0.8090±0.0158 | 90.37±2.30 |
| **C. 2** | 86.68±4.33 | 88.65±5.54 | 0.8222±0.0535 | 88.29±5.94 |
| **C. 3** | 94.57±1.06 | 97.44±0.73 | 0.9677±0.0048 | 97.90±0.74 |
| **C. 4** | 85.06±3.17 | 88.08±3.81 | 0.8148±0.0341 | 89.27±4.17 |

**Supplementary Table 7. Further Quantifications of the LSTM Model Performance in Decoding Characters.**In addition to the LSTM model F1-scores and accuracy values shown for all participants in Fig. 2e and Fig. 2f, here we report mean±STD values of other measures such as recall and precision to quantify model performance in decoding each of the characters (rows).

| Layers | | Feature Map | Kernel Size | Stride | Activation |
| --- | --- | --- | --- | --- | --- |
| Input | Neural Signal | N_neurons_*60*1 | - | - | - |
| 1 | Conv + BatchNorm | n_1_*60*12 | 1*1 | 1 | LeakyReLU |
| 2 | Conv + BatchNorm | n_2_*12*12 | 8*8 | 1 | LeakyReLU |
| 3 | Conv + BatchNorm | n_3_*10*10 | 3*3 | 1 | LeakyReLU |
| 4 | Conv + BatchNorm | 8 | 3*3 | 1 | LeakyReLU |
| 5 | MaxPooling | - | 3*3 | 3 |  |
| 6 | Flatten | Variable (see below)† | - | - | - |
| 7 | FC + BatchNorm | 64 | - | - | LeakyReLU |
| 8 | FC | 12 | - | - | Softmax |

**Supplementary Table 8. CNN Architecture.**The CNN model was used as an independent confirmation of the LSTM model results. Here, too, the windowed neural signal (firing rate of all neurons from a participant during a two-second interval around each frame) was the input that underwent several convolution layers with LeakyReLU activations and Batch Normalization (BatchNorm) layers in between. The output of the last convolutional layer went through a MaxPooling layer and was then flattened to a one-dimensional vector. This one-dimensional vector was concatenated with the flattened region tag embedding corresponding to each region in order to incorporate more information into the model. Finally, the concatenated vector was fed into two sequential Fully Connected (FC) layers with LeakyReLU activation and a Batch Normalization in between. The output of the last Fully Connected layer was reshaped into a 4*3 matrix corresponding to the confidence scores of the three labels (Yes, No, DNK) for each of the four main characters.

† Given that each participant had a different number of contacts, the number is variable from participant to participant.
